# Possible molecular and cellular mechanisms at the basis of atmospheric electromagnetic field bioeffects

**DOI:** 10.1007/s00484-020-01885-1

**Published:** 2020-04-25

**Authors:** Michal Cifra, Francesca Apollonio, Micaela Liberti, Tomás García-Sánchez, Lluis M. Mir

**Affiliations:** 1 Institute of Photonics and Electronics of the Czech Academy of Sciences, 18251, Chaberská 1014/57, Prague, Czechia; 2 Department of Information Engineering, Electronics and Telecommunications, Sapienza University of Rome, Rome, Italy; 3 Department of Information and Communication Technologies, Universitat Pompeu Fabra, Barcelona, Spain; 4 Université Paris-Saclay, CNRS, Gustave Roussy, Aspects métaboliques et systémiques de l’oncogénèse pour de nouvelles approches thérapeutiques, 94805, Villejuif, France

**Keywords:** Electromagnetic field, Bioeffects, Atmosphere

## Abstract

Mechanisms of how electromagnetic (EM) field acts on biological systems are governed by the same physics regardless of the origin of the EM field (technological, atmospheric...), given that EM parameters are the same. We draw from a large body of literature of bioeffects of a man-made electromagnetic field. In this paper, we performed a focused review on selected possible mechanisms of how atmospheric electromagnetic phenomena can act at the molecular and cellular level. We first briefly review the range of frequencies and field strengths for both electric and magnetic fields in the atmosphere. Then, we focused on a concise description of the current knowledge on weak electric and magnetic field bioeffects with possible molecular mechanisms at the basis of possible EM field bioeffects combined with modeling strategies to estimate reliable outcomes and speculate about the biological effects linked to lightning or pyroelectricity. Indeed, we bring pyroelectricity as a natural source of voltage gradients previously unexplored. While very different from lightning, it can result in similar bioeffects based on similar mechanisms, which can lead to close speculations on the importance of these atmospheric electric fields in the evolution.

## Introduction

Life evolved and persisted in the environment where, among other factors, it is permanently exposed to natural electromagnetic field (nEMF), often termed as natural electromagnetic background or electromagnetic noise. It is logical to ask whether this nEMF has or had any effect on biological systems (“bioeffect”) ranging from biomolecules to ecosystems. From the perspective of applied research, it is necessary to understand the biological effects of nEMF especially on the level of humans and potential health effects and well-being. On the one hand, if any negative or harmful effects were observed, measures would need to be taken to protect the population from unwanted exposure to nEMF phenomena or to reduce such negative effects. On the other hand, if the stable/optimal nEMF background turned out to be beneficial or even essential for life, human health, and well-being, it would present an important piece of knowledge, for example, for space travels. From the perspective of fundamental research, there is great importance to understand the mechanisms of biological effects, if any, of nEMF. Such an understanding would be necessary to form a theoretical basis to predict the nEMF bioeffects to prevent any potential negative effects and harness beneficial effects. For the fundamental research of mechanisms of biological nEMF, one can leverage a large body of research literature of several thousands of papers (http://ieee-emf.com/) on biological effects of artificial EMF, which are anthropogenically generated on purpose by technological means. The underlying principle that enables us to leverage these data is that the basic physics of EMF should be the same regardless of the natural or anthropogenic origin of EMF.

## Section 1: Parameters of the natural electromagnetic field

**Fig. 1:**
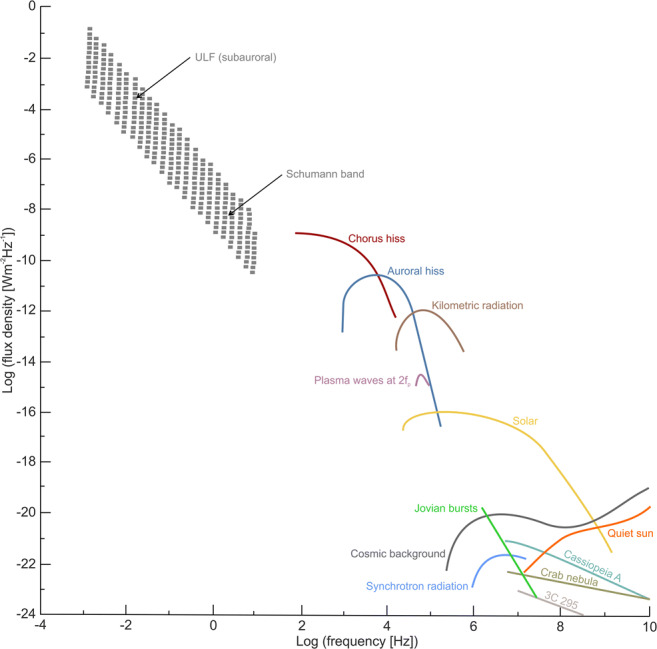
Spectrum of natural electromagnetic background, adapted from chapter 1 by Lanzerotti and Southwood from Parker et al. (1979)

In order to understand the potential biological effects of nEMF present on Earth, one first needs to know the basic parameters of nEMF: the range of frequencies and field strengths for both the electric field and magnetic field components. In this review, we focused on the frequency range from 0 Hz (static fields) to 300 GHz, the classical upper range of EMF frequencies. See Fig. 1 for spectral overview of selected nEMF phenomena.

Probably the most well-known nEMF phenomenon is the static magnetic field of the Earth. Electric currents in the conductive melted iron alloys in the Earth’s core are believed to generate this magnetic field (Kuang and Bloxham 1997; Weiss 2002). The shape of the Earth’s magnetic field can be approximated by a magnetic dipole, but there may be notable local deviations. In essence, the magnetic field strength on the Earth’s surface may have values in the range from ca. 25 to 70 μT. The strength and the actual shape of the Earth’s magnetic field fluctuates on time scale of milliseconds and hours (Hayakawa et al. 2004) to millions of years (McElhinny and McFadden 1998). In recent years, the lowest value of less than 25 μT is in South America and the highest values beyond 60–65 μT are in two locations: one close to Antarctica (on the connecting line to Australia) and one in central Krasnoyarsk region in Russia (http://www.ngdc.noaa.gov/geomag/WMM/data/WMM2015/WMM2015_Report.pdf). On the time scale of hundreds of years, the Earth magnetic dipole is decreasing: the magnetic field strength dropped by ca. 9% from 1840 (Hulot et al. 2002; Finlay et al. 2016)

The Earth’s atmosphere is also generating an omnipresent static electric field due to the global atmospheric electric circuit (GEC) (Rycroft et al. 2000). The field strength is close to 100 V/m under fair weather conditions to a few 10 kV/m during storms. The Earth surface and ionosphere represent spherical layer concentric conductors electrically connected by weakly conducting (leaky) capacitor created by the air gap. The electric potential difference between the ionosphere and Earth surface is ca. 200 kV and the effective resistance of the whole atmospheric air mass is around 200 Ohm, which gives about 1 kA current via Ohm’s law. The overall capacitance of the Earth capacitor is around 0.7 F, which together with the effective resistance yields to the time constant of ca. 2 min. The GEC has a maintained quasi-stationary current distribution, which is powered by the lightning strikes that arise due to charge separation inside electrified clouds.

In the following text, we focused on time-dependent nEMF phenomena, which we touched already by mentioning lightning strikes. One major determinant of the frequencies and corresponding wavelengths is the size of the electromagnetic Earth-related cavities—the general rule is that half of the wavelength (and its integer multiples) of the EMF wave has to “fit” in the cavity. The objects which create cavity boundaries are the Earth’s surface, the ionosphere, and the magnetosphere (Fig. 1 in Bianchi and Meloni (2007)). The magnetospheric cavity supports EMF modes in the ultra-low frequency range of 1–3000 mHz which are powered by the particles of solar origin and their radiative pressure on the magnetosphere. Ionosphere-Earth surface cavity supports various types of EMF modes in the frequency range from 3 Hz to 30 MHz (Tab. 1 in Bianchi and Meloni (2007)) and basically all of them are excited/powered by the lightning strikes since they cover this spectral range (Fig. 2 in Bianchi and Meloni (2007)). There are daily ca. 2000 storms worldwide with several million lightning strikes in total, which makes ca. 100 strikes/s worldwide. The single discharge can carry up to ca. 10 kA current and energy of ca. tenths of GJ.

These natural electromagnetic impulses are damped and of short duration (Panagopoulos and Balmori 2017). High voltage differences can be originated from the electric charge exchange inter-clouds, intra-clouds, from cloud top upwards, and between clouds and ground. When the voltage difference becomes sufficiently high, producing electric fields on the order of ≥ 10 kV/cm and at the same time the conductivity of the atmosphere increases due to the content of moisture during thunderstorms atmospheric discharges may occur. These are the classical conditions for spark discharges’ formation, where electromagnetic waves are generated and propagated through back-and-forth reflections from the lower part of the ionosphere and the Earth’s surface: electromagnetic guidance provided by natural boundaries. This continuous reflection of the electromagnetic waves creates in the Earth-ionosphere cavity excited by global lightning discharges extremely low frequency (ELF) electromagnetic resonances that can be measured in the range between 5 and 60 Hz, called Schumann resonances (Panagopoulos and Balmori 2017). The fundamental mode has a frequency between 7.5 and 8 Hz (Votis et al. 2018), which is directly related to the Earth circumference and velocity of light propagation (Williams et al. 2006). The detectable higher modes reach up to ca. 40 Hz and the corresponding field strength is in the pT range. Other nEMF phenomena, while being only transient in contrast to Schumann resonance, are radio atmospheric phenomena, so-called sferics. They persist only for a few milliseconds and their spectrum originates from a lightning strike and is modulated by the Earth-ionosphere waveguide, thus reaching up to a few tens of kHz.

Higher radiofrequency and microwave band as part of nEMF are far less prominent. The transient surge (during geomagnetic storms) of microwave emission (3–30 GHz) radiation from Rydberg excited states of O_2_ and N_2_ molecules at the higher ionosphere (with intensities up to 10^−11^–10^−12^ W/cm^2^ at the generation site) is probably the major nEMF microwave phenomenon originating in the Earth’s atmosphere (Avakyan and Voronin 2006). There are also cosmic sources of microwave signals, including cosmic background, Jovian bursts, and signals from distant cosmic objects such as Cassiopeia, Crab Nebula, and 3C 295 (Fig. 9 in Bianchi and Meloni (2007)), but the intensities incoming to Earth are very low, at about 10^−22^–10^−19^ W/(m^2^.Hz).

It is interesting to note that there are multiple mechanisms that couple the Earth lithosphere processes to atmosphere and ionosphere and GEC as such. For instance, microfracturing, liquid diffusion, pressure variations, water elevation, and gas releases change the air content and its conductivity, hence affecting the current flowing to the ionosphere (Hayakawa et al. 2004). GEC is not only coupled to lithosphere but is also influenced by extraterrestrial physical processes. The major class of solar-terrestrial effects on the GEC comes from galactic cosmic rays - GCR (high-energy corpuscular-charged particles), which vary spatially and temporally, and energetic electrons precipitating from the magnetosphere (Rycroft et al. 2012). The fact that GCR ionize the air and affect its conductivity is the reason why they are the major effectors on GEC. Interestingly, as the sun’s magnetic field varies during 11 (22) year solar cycle, it modulates the intensity of GCR incoming to Earth (Ross and Chaplin 2019). Importantly, even a few percent variation of GCR flux may affect the thunderstorm charging current and ionospheric potential (Siingh et al. 2007).

## Section 2: Molecular mechanisms at the basis of possible bioeffects combined to modeling strategies to estimate reliable outcomes

As mentioned in the previous section, electric fields present in the atmosphere are typically in the range of 120–150 V/m, although under specific conditions, they can reach values up to 10 kV/m, for example, near thunderstorms. This kind of fields does not vary with time and hence they are defined as static; they are generated between the positively charged ionosphere and the Earth’s negatively charged surface. Static electric fields are not able to penetrate the human body because of its high conductivity, and for the same reason, their displacement is always perpendicular to the body surface. It is assumed that a static electric field within the body is attenuated by a factor of about 10^−12^ from an external source. These fields induce a surface electric charge and when the strength of the field is sufficiently high, the induced charge may be perceived through its interaction with body hair. If the charge reaches a sufficiently high level, a corona discharge may occur (Repacholi and Greenebaum 1999). Such a perception threshold depends on different factors and can vary between 10 and 45 kV/m. Some evidence is present regarding the capability of both humans and animals to detect and respond to static stimulation (Petri et al. 2017). In particular, experiments on the perception of static electric fields showed evidence that detection thresholds are lower for whole-body exposure than that for limb exposure. Moreover, it has been suggested that hair movements due to electrostatic forces play an important role in this kind of perception. However, apart from this type of interaction, no other direct action of these fields on living systems is known. These outcomes have also been standardized by the World Health Organization (WHO) in the “Static Fields Environmental Health Criteria Monograph No.232,” where an international group of experts reported that the only adverse acute effects of static electric fields are associated with direct perception and discomfort from micro shocks (World Health Organization and others 2006).

Different is the case of the high-intensity electric fields generated during lightning. The role of lightning and atmospheric discharge in the evolution of life has been greatly suggestive, with a direct link to the natural horizontal gene transfer in bacteria (Kotnik 2013). The molecular mechanism, in this case, is related to electroporation: an electric pulse with amplitude and duration sufficient to induce membrane electroporation has been shown to allow nucleic acid penetration in the cells exposed (as presented in Section 3). However, to prove the hypothesis of a gene transfer mediated by atmospheric lightning, controlled exposure conditions are necessary (Liberti et al. 2013), as well as a combined approach integrating reproducible experimental data with numerical modeling aiming at understanding and interpreting biological results (Denzi et al. 2017). In particular, the readapting version of the Kitano cycle proposed for system biology (Kitano 2002). The basic concept is the possibility to perform “Model-Driven” and “Experiment-Driven” results, outlining the importance of continuous exchange between the biological results and the numerical modeling. This can be convincingly performed on a molecular level, where it is possible to perform modeling with atomistic details. For example, it was recently predicted via molecular dynamics simulations that a tubulin protein conformation can be affected by an intense electric field at the nanosecond timescale (Marracino et al. 2019). Consequently, it was demonstrated experimentally that indeed tubulin conformation in vitro can be modulated by intense electric pulses, and depending on the parameters of the treatment, the protein self-assembles to different structures (Chafai et al. 2019).

Another natural electromagnetic phenomenon associated with atmospheric nEMF is the electrical discharge and the related electromagnetic waves that are generated by lightning during a thunderstorm. As discussed in Section 1, these natural fields, consisting of short-duration and damped oscillating electromagnetic impulses, are able to affect the Earth’s environment and the living organisms (Panagopoulos and Balmori 2017). Despite one recent in vitro result of the influence of electromagnetic fields at the Schumann resonance in rat cardiomyocyte cultures (Elhalel et al. 2019) and some evidence that this kind of fields can be sensed by humans through different types of indications, such as headache and fatigue (Panagopoulos and Balmori 2017), up to now, a clear explanation for this association has not been provided.

Regarding the static magnetic field, the natural geomagnetic field of the Earth is around 50 μT and varies between values of about 35 and 70 μT, depending on the geographic location. Static magnetic fields have been mainly involved in the migratory behavior of certain animal species. According to the World Health Organization and others (2006) the assessed experimental evidence has made possible the classification of the physical mechanism of interactions of static magnetic fields with biological systems in three classes: (i) interaction with ionic conduction current; (ii) magneto-mechanical effects; (iii) effects on electronic spin states of reaction intermediates. Exposure to static magnetic fields will affect electrically charged particles and cells in the blood when moving through the field; for example, the field can reduce the velocity of blood cells flowing through blood vessels. However, only for magnetic induction fields exceeding 8 T, which are orders of magnitude larger than the nEMF, acute effects are likely to occur, ranging from minor changes in a heartbeat to an increase heart rhythm (arrhythmia) (Repacholi and Greenebaum 1999). For non-acute effects, outcomes related to human psychological, neurological, cardiovascular, immunological, and behavioral variations are controversial even if the rationale behind this association has been rigorously investigated (Close 2012). One possible hypothesis is related to the influence of cryptochrome on circadian rhythm in response to magnetic exposure. This kind of role could be related to radical-pair mechanism where the yield of a biochemical reaction might be sensitive to the orientation of an external magnetic field. Somewhat surprisingly, while magnetodetection in humans is not widely accepted, there is increasing evidence suggesting that such a sense may exist. In recent studies, it has been demonstrated that weak magnetic fields as the geomagnetic ones can provoke evoked potentials in humans (Carrubba et al. 2007) or can influence the visual sensitivity of man (Thoss and Bartsch 2007), supporting the evidence for the radical-pair retinal model in humans. Further experiments on magnetodetection have demonstrated, as the first thing, that cryptochrome is necessary for a light-dependent magnetic sense in Drosophila (Gegear et al. 2008) and successively that the human cryptochrome CRY2 has the molecular capability to function as a light-sensitive magnetosensor (Foley et al. 2011). In these last experiments, using a transgenic approach, it has been demonstrated that human cryptochrome can function as a magnetosensor in the magnetoreception system of the Drosophila in a light-dependent manner, opening the way to a renewed interest for human magnetoreception. As a whole, the conclusive recommendation of the WHO group of experts on static magnetic fields is that more research should be accomplished with in vitro studies, animal and volunteer experimental studies, and epidemiological studies (World Health Organization and others 2006).

**Fig. 2:**
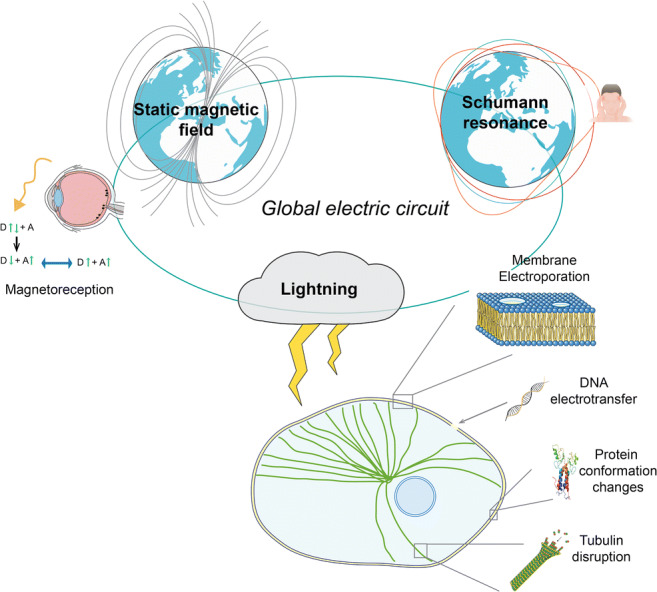
Representation of some of the possible bioeffects caused by atmospheric electromagnetic fields

As a final consideration, the only way to prove a possible effect of electromagnetic fields on human beings is to link experimental and modeling aspects. As a first step, a systemic approach should be adopted combining together all the positive studies to identify specific plausible targets and related pathways. Successively, a multiscale methodology should be used as reported (Apollonio et al. 2013). In fact, a biological effect can be considered as the ultimate step of a chain of events starting with the field interacting with a biological system at the level of a single molecule or structure, through the modification of its charge distribution, its chemical state, or its energy. The change provoked by the field at the molecular level can be sensed and reinforced across the complexity of the biological scale to produce a response of the whole organism. Multiscale approach, extending from the most basic of amino acid sequence that constitute protein function to concerted multicellular signaling cascades, thus stratifying different levels of biological organization, each with its own complexity, structure, and function, is the unique way to provide not only a scientific support to experimental evidence but also a useful interpretation of the results obtained. Figure 2 and Table 1 summarize the mentioned biological effects.

**Table 1:** Summary of selected EM biological effects mentioned in the manuscript highlighting physical origin of nEMF phenomenon, its respective parameter, nature of the effect observed, status of the acceptance of the effect by scientific community, and references

Physical origin	Parameter	Effect	Status	Reference
Static magnetic field	B [T]	Human effect: acute (> 8 T): increased heart rhythms	Assessed	Repacholi and Greenebaum (1999), World Health Organization, others (2006)
		Human effects: non-acute (< 8 T): human psychological, neurological, cardiovascular, immunological, and behavioral variations	Controversial based on hypothesis to be verified (magnetodetection)	Close J (2012), Thoss and Bartsch (2007), Gegear et al. (2008), Foley et al. (2011)
Static electric field	E [V/m]	Human effect: perception of static electric fields (threshold about 10–45 kV/m)	Assessed	World Health Organization, others (2006)
High intense electric field pulses of ns duration	E [V/m]	In vitro effect: Electroporation of cells	Assessed	Mir (2009), Breton et al. (2012), Azan et al. (2017)
		In vitro effect: Electrotransfer of genes	Assessed	Ceremonie et al. (2006), Kotnik (2013)
		Change in tubulin conformation	Evidence coming from model and experiments	Marracino et al. (2019), Chafai et al. (2019)
EM fields (low frequency) Schumann resonances (SR)	B [T] and/or E [V/m]	Human effect: headache, fatigue	To be ascertained	Panagopoulos and Balmori (2017)
		In vitro effect: Reduction in spontaneous mechanical contractions of cardiomyocytes	Experimental evidence	Elhalel et al. (2019)

## Section 3: Natural sources of voltage gradients in the atmosphere

Atmospheric electricity often refers to two major phenomena caused by the difference of potential between the lithosphere and the ionosphere, thus affecting the atmosphere. This potential difference results from charge generation by the interaction of cosmic rays and components of the solar wind with the upper layers of the atmosphere, the ionosphere. These two major phenomena, as already presented in Section 1, are the following: (a) an ultra-low frequency electromagnetic field (at about 7.8 Hz, the “Schumann resonance”) generated by the constant occurrence of lightning; that is, the electrical discharges occurring in the atmosphere; and (b) an atmospheric voltage gradient of an average amplitude of about 100 V/m extending between the ionosphere and the lithosphere. This voltage gradient of about 100 V/m displays a circadian rhythm with an amplitude of ± 30 to 40 V/m (sometimes more). But what is important to mention here is the fact that all the organisms living in the atmosphere are permanently submitted to this atmospheric electricity.

Organisms can also be exposed, accidentally, to the lighting itself. Lightning is a complex signal: besides the progression of a very high voltage difference (and high current) traveling through the atmosphere and ionizing it, lightning is accompanied by the emission of ultraviolet light and the production of radicals. This complex signal can be reproduced in the laboratory. Because the progression of the lightning is based on ultra-short steps (in the nanosecond range), it is also possible to strictly reproduce in the laboratory the “electrical” component of the lightning provided that the electric pulse generator provides ultra-short (subnanosecond or a few nanoseconds long) and intense (in the MV/m range) electric pulses. This can be achieved using various technological approaches (Lucia et al. 2019). These short intense electric pulses are known to affect the cell membranes and provoke their “electroporation” or “electropermeabilization,” which can be reversible or irreversible. Indeed, after the cell exposure to these pulses, the plasma membrane of the cells can become permeable to otherwise “nonpermeant” molecules (such as nucleic acids (DNA, RNA), charged hydrophilic molecules, some small drugs or dyes not actively pumped across the membrane…) (Mir 2009; Breton et al. 2012; Azan et al. 2017).

Reversible cell electroporation minimally affects cell viability because of the reversibility of the perturbation caused by the electric pulses. One of its current applications in vitro and in vivo in the laboratories is the electrotransfer of genes. In cuvettes or in animals, long or short sequences of DNA or RNA are brought in contact with the cells, and their uptake is made possible by the cell electropermeabilization. A study using conditions that were mimicking the situation of lightning striking a soil, with bacteria and fragments of DNA on the soil, demonstrated possible gene electrotransfer in nature. This study highlighted the possible impact of lightning on horizontal gene transfer between soil microorganisms and thus the potential role of this accidental exposure to electric fields in the evolution of these microorganisms (Ceremonie et al. 2006).

It is important to recall that any new piece of DNA incorporated in the cell will be actively transported through the cytoplasm and imported in the nucleus in the case of the eukaryotic cells, or simply located close to the chromosome in the case of the prokaryotic cells with no nucleus. In both cases, DNA “recombination” can then occur, in particular, as part of the normal and natural mechanisms of DNA repair (necessary to maintain the stable genetic heritage of the cells). Therefore, the new information coded by the electrotransfer red DNA can be added to the heritage of the cells and as such transmitted from generation to generation. In such a way, the phenomenon described in this paragraph can actually influence the evolution, allowing for the horizontal transfer of genes and the acquisition of new functions.

While the human activities produce variable electromagnetic fields in the atmosphere because of the use of radars and short- and long-range telecommunications, including mobile phones (not considered in this article), there are other sources of natural electricity (that is, generated without human intervention) to which organisms can also be discontinuously exposed. One of these sources, quite widespread, is the biogenic one, generated by living organisms, such as plants, insects (e.g., bees), and many other electrical living beings, even though the most known biogenic sources are in the hydrosphere (thus not in the atmosphere). Indeed, the salty seawater is very conductive and electric fields can convey long-distance information (e.g., the electrolocalization of electric fishes), while the air (in particular the continental dry air) is not conductive and does not permit such information channeling. The most known electrical animals are the catfish, the electric eel, and the torpedoes, which possess specialized electric organs (like the Sach’s organ or the Main and Hunter’s organ) used either to localize the prey or to catch it.

There is another well-known source of natural electricity that lies just at the surface of the lithosphere, that is, at the limit between the atmosphere and the lithosphere. Its origin is not biogenic but “geological,” and needs a physical perturbation to appear. The Saint Elm’s fires result from a perturbation of the atmospheric electricity, but similar events have been reported in areas subjected to very strong earthquakes just before or even at the time of earthquake occurrence. This telluric electricity is due to magma and crust movements. Recently, we explored the potential consequences of another “geological” source of “natural” voltage gradients, the pyroelectricity and its effects on microorganisms or cells.

## Section 4: Pyroelectricity, another natural source of voltage gradients

Many people feel familiar with the term piezoelectricity. Piezoelectricity results from the capability of certain crystalline materials to be spontaneously polarized when they are submitted to mechanical stress. In contrary, a controlled displacement of objects placed on top of piezoelectric materials can be achieved with submillimetric accuracy thanks to the application of electric pulses. Pyroelectricity is much less known. Pyroelectricity results from the capability of other crystalline materials (and also from non-crystalline materials such as bone, certain proteins, and DNA) to be spontaneously polarized when they are submitted to a change in temperature (Joshi and Dawar 1982). This change in polarization translates into a change of the dipole moment of the atoms/molecules: it leads to a redistribution of charges at the surface of the pyroelectric compounds. The consequence is the generation of an electric field (EF) at the surface of the pyroelectric materials if there is a change in temperature, whether “naturally” or because of the human beings’ activity. Moreover, the amplitude of the voltage gradient depends on the relative change in temperature (Lang 2005). Like piezoelectric materials, pyroelectric materials are found in nature. Crystalline structure favors the occurrence of these properties. Tourmaline is a natural borosilicate complex found under different forms. Especially, crystalline tourmaline particles of a few microns display a spontaneous and permanent dipole. To investigate possible biological effects of pyroelectricity, tourmaline microparticles have to be either in direct contact with the cells or at least in their vicinity. However, the physical presence of a mineral crystal close to the cell surface may already have biological effects on these cells. Therefore, to demonstrate the biological effects of pyroelectricity, tourmaline must be subjected to changes in temperature of different amplitude prior to their application to cells and the amplitude of the consequences on the cells must depend on the amplitude of the temperature changes to which the tourmaline nanoparticles are exposed.

As mentioned in Section 3, a relatively well-known effect of an electric field on the cell membrane is the change in the cell membrane permeability. Indeed, the exposure of cells to electric fields results in the induction of a change in the amplitude of the resting transmembrane potential of the cell membrane. In the above given field amplitude thresholds, which are very dependent on the duration of the exposure to the electric field (García-Sánchez et al. 2019), the transmembrane voltage (the net one, corresponding to the superposition of the resting one and the one induced by the electric field) causes changes in the membrane structure as well as in the conductivity of the membrane and in its impermeability to water and other hydrophilic molecules.

The changes in the permeability of the cell membrane to a non-permanent cytotoxic agent were assessed by a cloning efficacy test. The changes in the cell membrane permeability were studied in vitro, through the exposure of DC-3F mammalian cells in suspension at 24 °C in S-MEM to tourmaline (1 mg/ml, 3 μm particle mean size). Before its addition to the cells, tourmaline was stored at various temperatures: + 24 °C, + 4 °C, + 95 °C, and − 196 °C. Significant increases in cell membrane permeability were detected only if tourmaline had been previously subjected to a temperature change, suggesting that pyroelectricity could be involved indeed in the increase in the membrane permeability (García-Sánchez et al. 2018). As previously studied in detail, the exposure of cell membranes to electric pulses can increase their permeability to various types of molecules in a phenomenon known as “electroporation” or “electropermeabilization” and which results from reversible changes in the lipid bilayer.

In conclusion, using a classical test to reveal cell electropermeabilization, it was shown that the presence of tourmaline nanocrystals resulted in cell transient permeabilization, only if tourmaline nanocrystals had been exposed to a previous change in temperature, responsible for pyroelectricity generation in these crystals. These changes in temperature are frequent in the atmosphere. For example, they can be the consequence of intermittent direct exposure to the sun due to meteorological reasons such as partial cloud coverage. Thus, the pyroelectric-induced cell permeabilization could actually result from the natural voltage gradients generated in the tourmaline nanocrystals by the temperature changes to which these crystals were exposed. The pyroelectric microparticles (or macroparticles if we extend the hypothesis to more large conditions potentially found in nature) might generate microdomains where the transmembrane potential threshold for cell permeabilization might be reached. Nevertheless, at this stage, we cannot fully exclude that other phenomena differing from the electropermeabilization classical mechanisms could be involved in the results achieved.

Today, with soils resulting from the biogenic activity and supporting vegetal coverage, direct pyroelectric material exposure to sunlight is probably very restricted, but in the early times of life on earth, the phenomenon discussed here might have had implications on the biological evolution. Indeed, changes in temperature of pyroelectric crystals at the surface of the soil could be generated under specific circumstances, for example, the passage from the shadow to intense sun illumination (and vice versa), particularly before the constitution of the atmosphere that we know today. This pyroelectricity could then have had impacts on the life evolution, similar to the possible impact of lightning on horizontal gene transfer resulting from the electroporation of soil microorganisms (Ceremonie et al. 2006).

The environmental man-caused pollution was not taken into account in the manuscript. In particular, with respect to the extremely large panel of anthropogenic electromagnetic fields, and with respect to the extreme variety of effects of the nano/microparticle pollution not related to their pyroelectric properties, the effects of the nanoparticle environmental pollution *due to their pyroelectric properties* is really small. Indeed, the effects due to their pyroelectric properties should be minimal, because the pyroelectric nanoparticles will constitute a minimal fraction of the total nanoparticle content in the environmental pollution, and also because these pyroelectric nanoparticles will not be exposed to large temperature variations prior to their exposure to human beings. Therefore, the pyroelectric field will be almost null, as well as the effects related to this very small electric field.

## Conclusion

In this short review, we first focused on clarification of the parameters of natural electromagnetic background present on Earth in the context of mechanisms of how the electromagnetic field could affect organisms. Then, we covered selected molecular mechanisms that underlie electric and magnetic field biological effects. We concluded that a multiscale modeling approach integrating the molecular response to field coupled to cellular and tissue and whole organism level is crucial for a complete understanding of electromagnetic field bioeffects. In the end, we covered an underappreciated source of endogenous voltage gradients through pyroelectric microscale materials. We suggested that endogenous pyroelectric particles could have an impact on life evolution via natural electroporation it might mediate.
